# A chronic pro-inflammatory environment contributes to the physiopathology of actinic lentigines

**DOI:** 10.1038/s41598-024-53990-5

**Published:** 2024-03-04

**Authors:** Christine Duval, Emilie Bourreau, Emilie Warrick, Philippe Bastien, Stéphanie Nouveau, Françoise Bernerd

**Affiliations:** L’Oréal Research and Innovation, Aulnay Sous Bois, France

**Keywords:** Mechanisms of disease, Chronic inflammation

## Abstract

Actinic lentigines (AL) or age spots, are skin hyperpigmented lesions associated with age and chronic sun exposure. To better understand the physiopathology of AL, we have characterized the inflammation response in AL of European and Japanese volunteers. Gene expression profile showed that in both populations, 10% of the modulated genes in AL versus adjacent non lesional skin (NL), i.e. 31 genes, are associated with inflammation/immune process. A pro-inflammatory environment in AL is strongly suggested by the activation of the arachidonic acid cascade and the plasmin pathway leading to prostaglandin production, along with the decrease of anti-inflammatory cytokines and the identification of inflammatory upstream regulators. Furthermore, in line with the over-expression of genes associated with the recruitment and activation of immune cells, immunostaining on skin sections revealed a significant infiltration of CD68+ macrophages and CD4^+^ T-cells in the dermis of AL. Strikingly, investigation of infiltrated macrophage subsets evidenced a significant increase of pro-inflammatory CD80+/CD68+ M1 macrophages in AL compared to NL. In conclusion, a chronic inflammation, sustained by pro-inflammatory mediators and infiltration of immune cells, particularly pro-inflammatory M1 macrophages, takes place in AL. This pro-inflammatory loop should be thus broken to normalize skin and improve the efficacy of age spot treatment.

## Introduction

A major and common hallmark of skin photoaging is the development of hyperpigmented disorders which have a negative psychosocial impact on people worldwide, and especially in Asia, where pigmented spots are the most important skin ageing features^1^. Among them, actinic lentigines (AL) also known as age spots or solar lentigines, are benign brown macules from a few millimeters to more than a centimeter in diameter. They appear with age, on the body skin exposed to sun (face, dorsum of hand, forearms) since their development has been associated with cumulative and intermittent UV exposure^2–4^. Beyond UV, air pollutants may also contribute to the development of facial lentigines^5,6^. Often multiple, they gradually increase in size over time. Thus, the demand for efficient and sustainable treatment is strong and first requires a better understanding of the physiopathology of these lesions.

We and others have shown that AL are characterized by alterations of the whole skin structure including epidermis, dermal epidermal junction and dermis^7–14^. Histologically, AL display deep epidermal invaginations that form bud-like or club-shaped elongations into the dermis. Melanin content is increased in the epidermis, mostly in the basal layer, along with a massive accumulation of melanin within the epidermal invaginations. Melanin excess cannot directly be explained by the activation of melanogenesis because of the discrepancy among results from different studies, some showed upregulation of melanogenesis-related genes in AL^10,15–17^ whereas others described no modulation of such genes^12–14^. Moreover, although melanocytes could be more dendritic^18^, their density along the dermal epidermal junction has been found similar in both lesional and perilesional skin, suggesting that their homeostasis remains physiological.

Interestingly, multiple molecular alterations have been revealed in AL. Dysregulation of keratinocyte proliferation and differentiation, and modifications of the dermal extracellular matrix (ECM) have been notably pinpointed and suggest a role of the keratinocyte homeostasis along with the dermal compartment in the physiopathology of lentigines^10–14,19^.

In two transcriptomic studies performed on AL clinically selected through dermoscopy on the hands from European or Japanese women, we strikingly discovered that, among modulated genes, one of the most prominent groups was related to inflammation/immunity response. Up to date, sparse information is available about this function in AL. Aoki et al^10^ succinctly noted the upregulation of three genes related to inflammation and 12 fatty-acid metabolism-related genes involved in the pathway of arachidonic acid synthesis. Goyarts et al^20^, through the upregulation of 6 genes associated with the inflammatory response and matrix metalloproteinases, predicted a local micro-inflammation in age spots. However, the robustness of these results remained uncertain since they were generated from a pool of RNAs of only three volunteers.

Recent histological studies mainly documented the abnormality of microvasculature in AL, showing a significant increase of the size and the volume of dermal blood vessels, as well as an augmentation of vessel branches with irregularly oriented microvessels^21–23^. Non-invasive OTC angiography also revealed an increased density of the blood vessels in facial solar lentigo^24^. Blood vessels are key in inflammation since they facilitate the influx of inflammatory cells. Indeed, CD68+ or CD163+ melanophages were noticed in the dermis of AL^22,25–27^. Furthermore, CD68+ macrophages were found densely gathered in regions with a high degree of branching vessels suggesting that the immune cell infiltration might be related to the increased vasculature of solar lentigo^23^. In turn, the presence of Vascular Epidermal Growth Factor (VEGF)-expressing macrophages in the perivascular areas indicates that macrophages could play a role in increased vasculature and inflammation in AL^22^. However, controversy remains regarding a significant increase of CD68+ macrophages in AL versus NL^22,25^.

Therefore, it is obvious that robust information on macrophages and on the diverse other immune cells (e.g. neutrophils, dendritic cells, mast cells, T-cells), critical to orchestrate the skin inflammatory/immune response is lacking. Notably, macrophages are plastic innate immune cells whose phenotype strongly depends on the local environment signals^28^. Although, in situ, a continuum of different phenotypes and functional states may exist, the M1/M2 paradigm introduced by Mills et al.^29^, described two well-established polarized types referred as classically activated M1 macrophages and alternatively activated M2, displaying specific and opposite functions along the inflammatory process. During the acute phase of inflammation, tissue damage or infection activates macrophages towards pro-inflammatory M1 macrophages associated with excretion of pro-inflammatory markers, a high level of antigen presentation and bactericidal/tumoricidal activity through the production of elevated quantity of toxic intermediates. Later, M2 macrophages mitigate inflammatory process by the expression of anti-inflammatory markers, promote resolution of inflammation and return to skin homeostasis by clearing tissue debris and apoptotic cells, stimulating angiogenesis and wound healing/repairing^29–31^. A thorough characterization of macrophages is thus required to better understand the inflammatory physiopathology of AL.

Therefore, the objective of this study was to better decipher inflammation in AL. First, a deep analysis of the inflammation/immune molecular signature, extracted from our previous full genome transcriptomic data on European and Japanese studies, was performed to compare AL and adjacent non-lesional skin (NL). Then, a precise in situ characterization and quantification of a panel of skin immune cells, particularly subsets of macrophages, were undertaken in AL and NL skin sections of the same studies.

## Results

### The molecular signature of AL predicts a pro-inflammatory environment and commitment of immune cells

Previous transcriptomic analyses from two independent clinical studies performed on European and Japanese volunteers revealed 245 differentially expressed genes between AL and NL, thus representing the common molecular signature of AL^14^. Genes were classified into functional families using a targeted bibliographic analysis focused on skin biology. One of the main represented functions was linked to inflammation & immunity^14^.

To understand the alterations of this function, a deeper analysis revealed a list of 31 genes significantly modulated in the same way in the two studies (Table 1), representing more than 10% of the total modulated genes. These genes belong to different subfamilies: inflammation/immune response, host defense and blood vessel regulation. Modulations of representative genes from each subfamily were validated using quantitative PCR analysis (qPCR) (Table S1). For each volunteer, very similar levels of gene modulation were obtained between qPCR and micro-arrays analysis, in both studies, confirming the robustness of the micro-array signature.

**Table 1:**
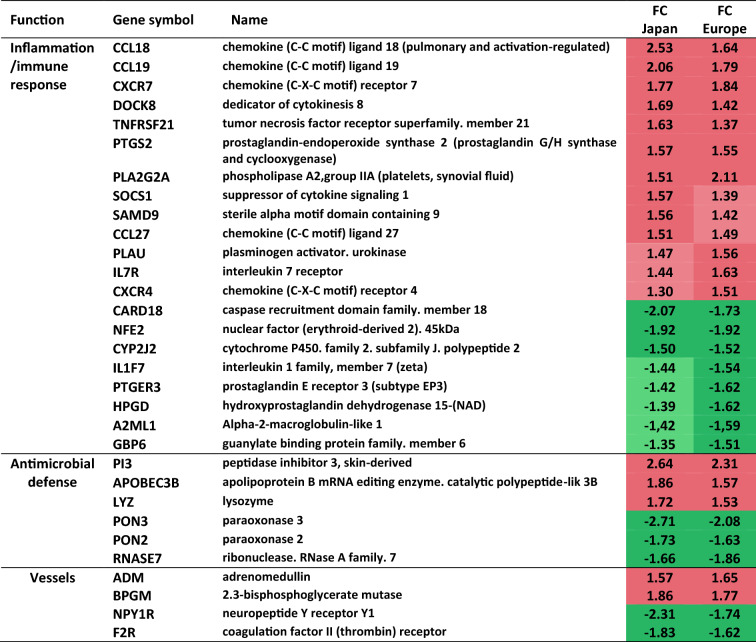
Inflammation/immunity-related genes modulated in actinic lentigos (AL) compared with non-lesional (NL).

31 genes belonging to the inflammation/immunity functional family were found significantly up-regulated (red)- or down-regulated (green) in AL versus NL samples in the two independent studies, European and Japanese. FC, mean fold change. Genes were identified from the 245 common genes significantly modulated in both studies. The list of 245 genes corresponds to the 178 probesets (136 genes) significantly modulated in the same way in AL in both studies with an absolute mean FC ≥ 1.5, completed with probesets modulated in the same way in both populations (109 genes), with an absolute mean FC ≥ 1.5 in one study and between 1.25 and 1.5 in the other (*p* value < 0.05)^14^. Then, genes were classified into the inflammation/immunity functional family trough a bibliographic study using Pubmed tools (http://www.ncbi.nlm.nih.gov/pubmed), with keywords related to skin, inflammation and /or immune cells.

A detailed analysis of inflammation/immune response subfamily revealed the modulation of genes exerting pro-inflammation, chemotaxis and activation of immune cells in AL versus NL. Indeed, a set of genes coding for enzymes, *PTGS2*, *PLA2G2A*, *CYP2J2*, *HPGD*, and a prostaglandin E2 (PGE2)-receptor *EP3*, favors and sustains, through the activation of the arachidonic acid (AA) cascade, the production of the potent pro-inflammatory and chemotactic mediator PGE2 in the lesional tissue. Moreover, upregulation of *PLAU/*PLASMINOGEN ACTIVATOR and downregulation of *A2ML1/*PLASMIN INHIBITOR ANTI-Α-2-MACROGLOBULIN-LIKE may conduct to the activation of plasmin pathway. Additionally, negative regulators of inflammation such as the interleukin *IL1F7*/IL-37 and the caspase inhibitor *CARD18*/ICEBERG were found downregulated. Interestingly, we observed the upregulation of numerous genes, *CCL18*, *CCL19*, *CCL27*, *CXCR7*, *CXCR4*, *TNFRSF21*, *IL7R*, *SOCS1*, *DOCK8*, *SAMD9* and *PLAU*, coding for chemokines, receptors or intracellular signaling factors associated with the recruitment and activation of immune cells such as macrophages and T-cells. In parallel, several genes such as *ADM, BPGM, NPYR, F2R/PAR-1*, related to blood vessels known to be tightly linked to the infiltration of immune cells, were also found modulated.

Of interest, we found the modulation of genes coding for potent antimicrobial peptides such as *RNASE7, PON2, PON3* (downregulated) and *APOBEC3B, LYSOZYME, PI3* (upregulated) which may contribute to the dysregulation of the innate cutaneous host defense in AL.

Additionally, using Ingenuity Pathway Analysis software (IPA), we identified upstream regulators that can explain, in cascade, the observed gene expression changes in our dataset. Significantly activated upstream regulators (z score > 2, overlapping *p* value < 0.01) are reported in Table 2. Among them, we particularly found three units of the NFkB pathway (NFkB complex, transcription factor NFKB1 and kinase IKBKB) as well as the transcription factor TNF, the cytokine IL-6 and the TLR7/8 receptor which are all known to be key actors of inflammatory/immune signaling. The numerous genes in the dataset targeted by NFKB complex (n = 15), IL-6 (n = 20) and TNF (n = 64) are shown in Figure S1.

**Table 2:**
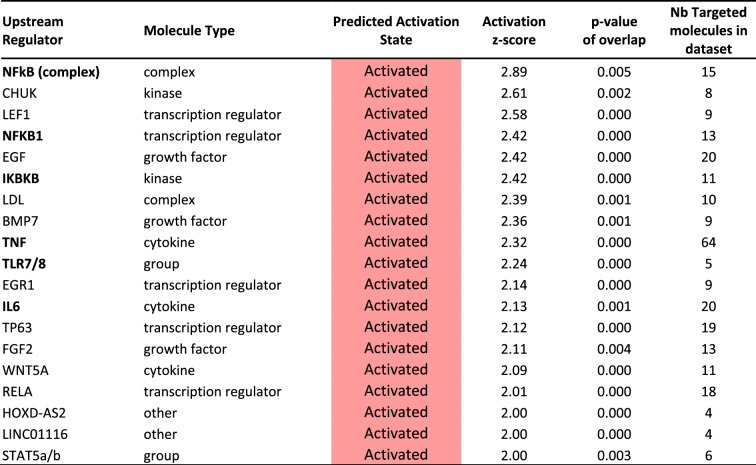
Upstream regulators identified by Ingenuity Pathway Analysis (IPA) from the list of 245 common genes significantly modulated in AL versus NL in both European and Japanese studies.

Upstream regulators with a predicted activation state (all z-scores were > 2.0) and a significant overlap *p* value of < 0.01 (Fisher’s Exact Test) are shown. Among the 19 found, 6 molecules/complex are involved in inflammation/immune signaling (in bold).

Altogether these data support a pro inflammatory context in actinic lentigo compared to the adjacent non lesional skin and suggest a potential infiltration and/or activation of immune cells in AL.

### Increased immunoreactivity of CD68 and CD4 reveals infiltration of macrophages and T-cells in the dermis of AL

To characterize the profile of immune cells subsets in actinic lentigo lesions, an immunostaining analysis was first performed on skin sections from biopsies from the European study for which we had at disposal biopsies dedicated to histology from more volunteers (n = 14) than for the Japan study (n = 8) (see Methods section).

**Figure 1:**
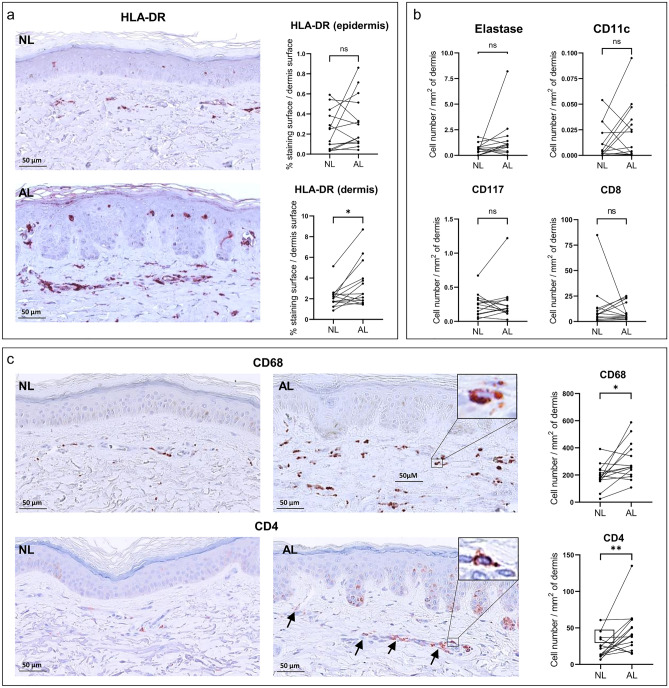
Immunostaining of inflammatory/immune cells in sections of AL and NL from European volunteers. (**a**) Representative illustration of HLA-DR staining in AL and NL sections of one volunteer, and plots of quantitative analysis of HLA-DR staining surface on skin sections of the epidermis and the dermis for each volunteer (n = 14). (**b**) Plots of elastase, CD11c, CD117 or CD8 positive cells quantified by image analysis in the dermis of AL and NL for each volunteer (n = 14). (**c**) Representative illustration of CD68 and CD4 staining in AL and NL sections of one volunteer and plots of CD68 and CD4 positive cells quantified in the dermis for each volunteer (n = 14). Inserts show high magnification of one CD68+ or CD4+ cell from black rectangles. Scale bars: main image 50 µm, insert 10 µm. Statistic comparisons between NL and AL groups were carried out using paired Wilcoxon signed ranks test: *p < 0.05; **p < 0.01; ns: non-significant difference. HLA-DR staining was significantly increased in the dermis of AL versus NL. No significant modulation of the number of elastase, CD11C, CD117 and CD8 positive cells was found. A significant increase of the number of CD68 and CD4 positive cells was revealed in the dermis of AL versus NL**.**

To have a first global view, HLA-DR antibody was used to identify antigen presenting cells. A strong labeling was observed in the epidermis and the dermis of AL and NL skin sections. The quantification of the staining revealed no significant difference of the stained surface in the epidermis of AL versus NL. However, an intense immunoreactivity and significant increase of the HLA -DR staining, around 1.5-fold was obtained in the dermis of AL versus NL (Fig. 1a). These results confirmed the interest to further investigate the immune subsets in AL particularly in the dermis.

Characteristic markers of neutrophils (Elastase), mast cells (CD117), dendritic cells (CD11c) or cytotoxic T-cells (CD8) were then studied. The number of cells expressing these markers was not found statistically different in the dermis of AL and NL (Fig. 1b).

In contrast, when the number of CD68+ macrophages and CD4^+^ T-cells was examined, we found a significant increase in AL versus NL (Fig. 1c). CD68+ cells were found dispersed throughout the dermis whereas CD4^+^ cells were more localized at proximity of the vascular structures**.** For CD68+ cells, the fold change was 1.96 (*p* = 0.013) in AL compared to NL, and 2.66 (*p* = 0.009) for CD4+cells (Table S2).

The results for these two positive subsets, CD68+ and CD4^+^ cells in AL from the European volunteers, led us to perform the similar analysis on the sections of biopsies from the Japanese study. The data confirmed that a significant increase of the CD68+ cells number/mm^2^ (mean fold change of 1.35, *p* = 0.008) as well as the number of CD4^+^ cells (mean fold change of 1.87, *p* = 0.018) was found in AL versus NL of Japanese volunteers (Fig. S2, Table S2).

### Increase of CD80+/CD68+ pro-inflammatory macrophages subset in the dermis of AL

To better characterize the infiltrated macrophage subsets, double immunostainings in situ were carried out. Co-detection of pan-macrophage CD68 antibody with commonly used CD80 antibody was made to assess pro-inflammatory M1. Similarly, a co-labelling of CD68 with antibodies against CD209 (DC-SIGN) or CD163, was performed to document anti-inflammatory/repairing M2 macrophages^32–34^.

**Figure 2:**
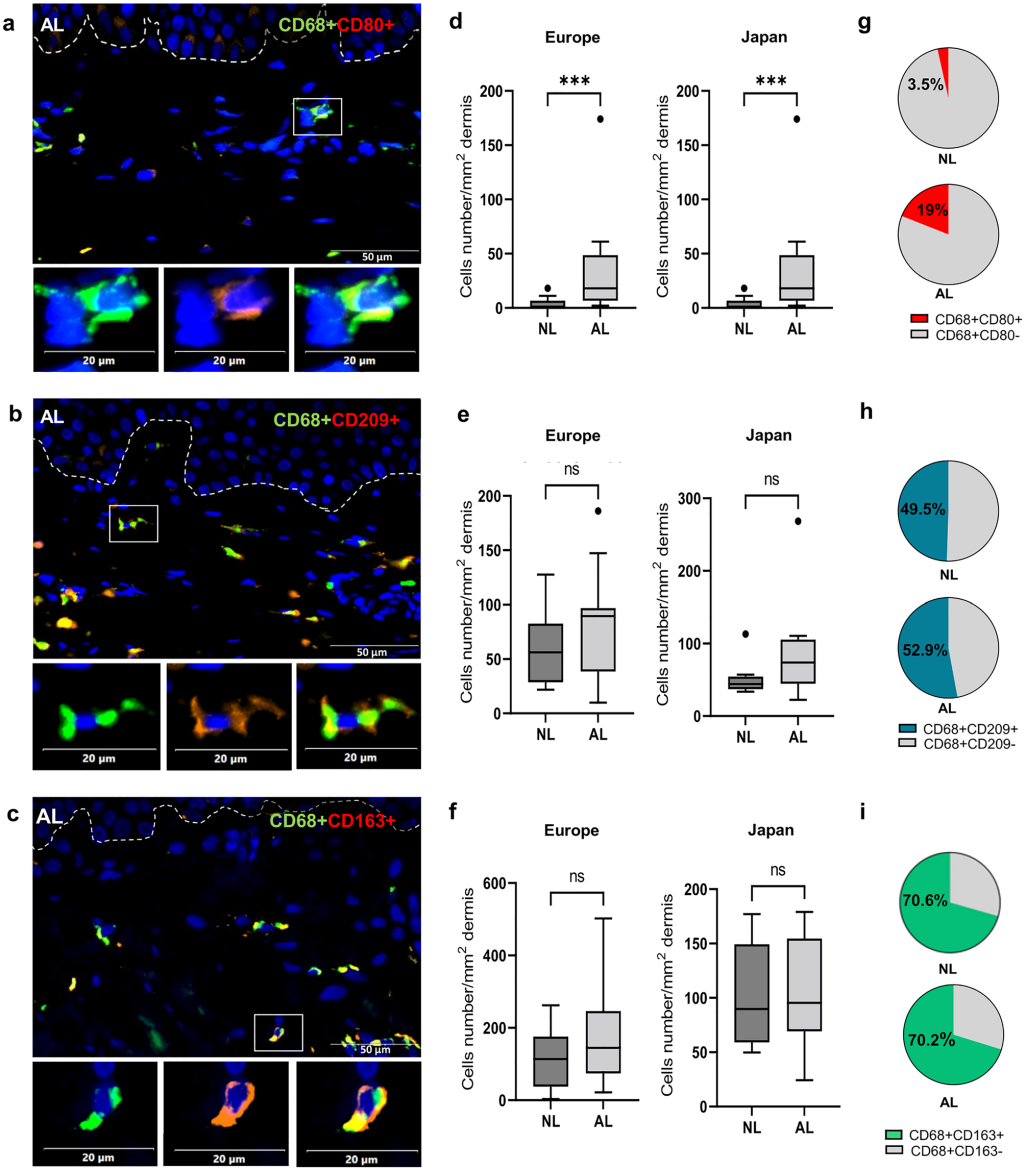
Identification and comparison of macrophages subsets in AL and NL skin sections. Representative illustration of AL section co-stained with antibodies against CD80 and CD68 (**a**), CD209 and CD68 (**b**), and CD163 and CD68 (**c**). Scale bar 50 µm. High magnification images of one cell below show CD80, CD209 or CD163 expression in red (left), CD68 in green (middle) and co-expression in merge images (right). Scale Bar 20 µm. Boxplots show the quantifications of the number of cells co-expressing CD80 and CD68 (**d**), CD209 and CD68 (**e**), and CD163 and CD68 (**f**) in the dermis of AL and NL, for the European (n = 14) and Japanese volunteers (n = 8). Statistic comparisons between NL and AL groups were carried out using paired Wilcoxon signed ranks test: ***p < 0.001; ns: non-significant difference. Pie charts represent the proportion of CD80+/CD68+ cells (**g**), CD209+/CD68+ cells (**h**) and CD163+/CD68+ cells (**i**) within the total CD68+ macrophages, expressed in %, in NL and AL (combined results of the European and Japanese studies). Nb: a fraction of CD68+ cells may co-express CD209 and CD163. They would be therefore reported in the two pies (**h**,**i**), explaining why the sum of the subset percentages appears superior to 100% (see results section). The results show the presence of the three macrophage subsets in AL and NL with an increase of CD80+/CD68+ cells in AL versus NL but no modulation of the two other subsets.

CD80+/CD68+, CD209+/CD68+ and CD163+/CD68+ cells could be observed dispersed throughout the dermis of AL and NL. A representative illustration of each immunolabelling in AL is shown in Fig. 2a–c. Quantification of CD80+/CD68+ cells in the dermis showed that in NL sections, the density of the cells was low in both studies with a mean of 3 ± 5.6 and 16 ± 14 cells per mm^2^ in European and Japanese volunteers respectively (Fig. 2d; Table S3). In contrast, more CD209+/CD68+ cells, with a mean of 60 ± 40 and 52 ± 35 cells per mm^2^, and more CD163+/CD68+ subsets, with a mean of 113 ± 84 and 102 ± 48 per mm^2^, were observed for European and Japanese samples respectively (Fig. 2e,f; Table S3).

Interestingly, when we compared the number of cells/mm^2^ in AL versus NL, the CD80+/CD68+ population was then found significantly more elevated both in AL from European and Japanese volunteers, whereas no significant modulation of CD209+/CD68+ and CD163+/CD68+ subsets was observed (Fig. 2d–f; Table S3).

Then the proportion of these macrophage subtypes within the global macrophage population has been estimated for each section, by calculating the percentage of the number of double positive cells (CD80+/CD68+ or CD209+/CD68+ or CD163+ /CD68+) versus the total number of CD68+ macrophages. The percentage means obtained for AL and NL sections of the 22 subjects included in the two studies (14 European and 8 Japanese) are reported in the pie charts (Fig. 2g–i). Strikingly, the pies in Fig. 2g show that the overall proportion of CD80+/CD68+ population in the two studies, representing 3.5% of the total CD68+ cells in NL, increased up to 19% in AL, indicating the presence of 5.4 more CD80+/CD68+ cells per mm^2^ in AL than in NL skin. On contrary, the change of the proportion of CD209+/CD68+ population in AL versus NL is very modest (52.9% vs. 49.5%) and absent for CD163+/CD68+ cells (70.2 vs. 70.6%) (Fig. 2h,i). A fraction of CD68+ macrophages co-expressing CD163 and CD209 markers may exist in skin sections and thus would be included in the proportion of both CD163+/CD68+ and CD209+/CD68+ subtypes (Fig. 2h,i), explaining why the total sum of the macrophages subtypes percentages can be over 100%.

## Discussion

We previously showed that actinic lentigines selected according to the similar clinical criteria by dermoscopy share comparable morphological and molecular alterations, notably the accumulation of melanin, abnormalities of epidermis homeostasis and dermal ECM organization^13,14^. As partially addressed by Aoki et al.^10^ and Goyart et al.^20^, we noticed another main represented altered gene expression family linked to “Inflammation & Immunity”. In the present study, a comprehensive analysis combining transcriptomic and immunohistochemistry, reveals that a specific pro-inflammatory molecular profile and an alteration of the innate and adaptative immunity response characterize AL compared to non lesional skin. Although the limited number of samples may represent a weakness of this research, the fact that the inflammatory molecular and histological profile of AL was found similar in two independent studies supports robustness of the results.

Indeed, the modulation in the same way of a large set of genes (n = 31) linked to “Inflammation & Immunity” in AL, together with the activation of the upstream regulators NFKB, TNF, IL6 and the IFNɣ signaling-inducer TLR7/8, attests that pathways known to foster a pro-inflammatory microenvironment were upregulated.

Particularly, a group of genes coding for enzymes involved in the activation of the arachidonic cascade and consequently in the production of the potent pro-inflammatory mediator PGE2, was found modulated. More precisely, the upregulation of *PTGS2*/COX2 and *PLA2G2A* leads to the metabolism of arachidonic acid into prostaglandins including PGE2. In addition, the downregulation of *CYP2J2* (cytochrome p450 arachidonic acid epoxygenase), involved in the catabolism of arachidonic acid into epoxy eicosatrienoic acids favors the prostaglandin production, whereas the downregulation of *HPGD* coding for the enzyme that degrades PGE2, sustains the PGE2 level. Altogether, these data suggest an increase of the prostaglandin level, notably of PGE2, within the lesional tissue. As the *EP3* gene which is the cAMP-inhibitory receptor of PGE2, was found downregulated in AL versus NL, an activation of the pro-inflammatory sequences induced by PGE2 -cytokines production, chemotaxis/differentiation of immune cells or vascular permeability- is thus favored through other PGE2 stimulating receptors^35^. Additionally, the activation of plasmin pathway, driven by the upregulation of *PLAU* and the downregulation of *A2ML1* gene, may participate in the reinforcement of inflammatory signals, such as the AA cascade activation, the immune cells and angiogenesis stimulation, along with the matrix degradation through the upregulation of metalloproteinases^36,37^. Even more, two genes, *IL1F7 (*coding for IL-37*)* and *CARD18* (coding for the caspase inhibitor ICEBERG), known to suppress inflammatory signals mediated by the NFkB pathway and to terminate inflammasome activation^38,39^, were found downregulated. This means that the potential of these two anti-inflammatory proteins to dampen the progression of inflammation in AL is decreased.

Besides, dysregulation of the innate defensive first line in AL is attested by the modulation of genes coding for proteins with highly potent antimicrobial properties such as *RNASE 7, PON2, PON3 APOBEC3B, LYSOZYME*^40–43^ and, notably, with the upregulation of *PI3* coding for ELAFIN, a serine protease inhibitor highly induced in keratinocytes of inflamed skin^44^. The downregulation of antimicrobial genes and others such as *IL1F7*, *CARD18* and *A2ML1*, normally expressed in the suprabasal layers of the epidermis, may be correlated to the impairment of epidermal differentiation in AL^10,13,14^.

The aforementioned modulated genes and a group of ten more, *CCL18*, *CCL19*, *CCL27*, *CXCR4*, *CXCR7*, *TNFRSF21*, *IL7R*, *SOCS1, DOCK8* and *SAMD9*, are well known to exert immunomodulatory activities by regulating inflammatory/immune cells from the innate and adaptative response. The CCL18, CCL19 and CCL27 chemokines, potentially increased in AL and previously linked to chronic skin inflammatory diseases^45,46^, could lead to the chemoattraction of macrophages and/or lymphocytes in AL. The activation of TNF signaling and the downregulation of *EP3* gene in AL, may conduct to the upregulation of *CCL27* in keratinocytes as CCL27 production is known to be specific of these cells^47,48^. The upregulation of *CXCR4* and *CXCR7*, coding for two cognate receptors of CXCL12/SDF1, may be also associated with chemotaxis and activation of macrophages and T-cells as well as vascular branching-morphogenesis^49–51^. *TNFRSF21*, *IL7R*, *SOCS1*, *DOCK8*, *SAMD9* are other important genes known to sustain the migration, homeostasis and integrity of T-lymphocytes^52–56^.

Therefore, these molecular changes highlighting multiple and relevant markers of immune cellular subtypes, prompted us to better characterize the cutaneous leukocytes population in AL. By histology, we first showed the absence of neutrophils, which are the first defense cellular line of the innate immune system, indicating that no acute inflammation takes place in AL. Mast cells, CD11c+ dendritic cells and CD8+ T-cells were not modified in AL versus NL suggesting that these cells are not to be involved in the physiopathology of AL. We did not explore epidermal Langerhans cells since the HLA-DR staining was not found significantly modulated in epidermis.

In contrast, we evidenced an increase of CD68+ macrophages and CD4^+^ cells (helper T-cells) in both the European and Japanese studies. Dermal CD68+ macrophages could not correspond to melanophages since melanin accumulation, previously described in the epidermis in these studies^13,14^, was not detectable in dermal cells (Fig. S3). The infiltration of CD68+ cells is in line with previous studies^22,23^ but for the first time, the modulation of immune cells involved in the adaptative response was demonstrated. These findings resonate with the modulation of the various genes in AL described above to stimulate recruitment and activation of macrophages and T-cells. This infiltration can be supported by the alteration of vasculature which controls immune cells extravasation already described in AL^23,24^. It is also in line with the modulation of blood vessels-associated genes found in our study; notably the upregulation of adrenomedullin, a potent proangiogenic and vasodilator molecule and previously found increased in lentigo^57,58^ and the downregulation of the *F2R*/PAR1 thrombin receptor, which could be a consequence of the vasodilatory prostaglandins (PGI2/PGE2)^59^, can be pinpointed.

It is well established that macrophages play a key role in orchestrating initiation, progression and resolution of inflammation, through pro-inflammatory or anti-inflammatory/proresolving functions according to their polarization (See introduction). The deeper characterization of the subtypes of macrophages in the dermis of AL and NL showed that the number of pro-inflammatory M1 (CD68+/CD80+) was increased in AL. Interestingly, the inflammatory molecular footprint makes sense with regards to this gain of M1 macrophages in AL. Particularly, NFkB is a key transcription factor related to M1 macrophage activation^30,60^. The upregulation of CCL19 which specifically induces the chemotaxis of M1 and not that of M2^61^, along with the decrease of *IL1F7*/IL37 and PON2, two molecules promoting the M1 to M2 polarization, may thus favor a M1 macrophage state in AL ^38,62^. Besides, the COX2/PGE2 axis stimulation observed in AL could be a trigger as well as a hallmark of macrophage polarization to pro-inflammatory M1 type^63^.

Two populations of M2 macrophages have been investigated through CD68/CD209 and CD68/CD163 co-labeling. The C-type lectin CD209 characterizes pro-resolving M2a macrophages^33^. The CD163 scavenger receptor is expressed by anti-inflammatory/remodeling M2c involved in tissue repairing functions^34^. No significant change in M2 populations was observed in AL versus NL. Noteworthy, the density of these cells, notably the CD163+/CD68+ ones, was found high in NL. This may be due to the fact that in photoaged skin, tissue damage has already occurred (e.g. solar elastosis) supporting mobilization of cells tempting to repair. Another explanation could be that CD163+ /CD68+ cells gather diverse M2 subpopulations and particularly a fraction of CD68+/CD209+ cells since, in vivo, CD163+ cells may also express CD209 as demonstrated by Fuentelsaz-Romero et al.^64^. Thus, to better decipher the macrophages phenotypes, pro-resolving versus pro-remodeling macrophages, a dedicated study with multiple co-staining would be necessary.

Photoaged skin itself displays inflammation and alteration of the immune cells’ pattern and function (inflammaging). Inflammatory infiltration of CD68+ macrophages, mast cells and CD4^+^ T-cells, as well as a decrease of CD8+ T-cells have been described in chronically sun-exposed skin^65^. Horiba et al. described a trend for an increase of CD86+ /CD68+ M1 macrophages, and a significant decrease of CD206+/CD68+ M2 macrophages^66^. We here show that in AL, the inflammatory molecular and cellular profile is different and even greatly aggravated compared to that of the adjacent photoaged skin.

Persistence of inflammation occurs in a tissue if the initiating stimulus is not removed or if the active programmed resolution of inflammation fails. What could be the inflammatory stimuli persisting in AL? By analogy with psoriasis, dysregulated keratinocytes may release cytokines and chemokines recruiting immune cells ^67,68^. An impact of the overcharge of melanin in keratinocytes would be possible as COX-2 expression and PGE2 production are induced during phagocytosis of melanosomes by keratinocytes^69^. Besides, the drastic alteration of the dermal compartment evidenced in AL may be implicated^13,14^; it has been proposed that immune cells become trapped within stiffer and cross-linked ECM matrices of aged tissues, contributing to ineffective orchestration of the immune response^70,71^. Additionally, senescent/aged fibroblasts, increased in AL^72^, characterized by the secretion of pro-inflammatory cytokines^73,74^ have been shown to drive the expression of a pro-inflammatory M1 phenotype of monocytes^75^.

In turn, the accumulation of pro-inflammatory M1 macrophages may have a detrimental impact on the lesional tissue by promoting inflammation progression, leucocyte recruitment and tissue degradation as these cells produce various pro-inflammatory cytokines and chemokines (TNFα, IFNγ, IL-1β, IL-6, IL-8, IL-12, IL-23, CCL2, CCL5, CXCL-5,9,10,11…), high level of toxic intermediates (nitric oxide, reactive oxygen species) as well as ECM-proteolytic enzymes^33,63,76–79^. Particularly, it was shown that M1 macrophages induce in dermal fibroblasts, a proinflammatory and ECM-degrading profile and enhance senescence^66,75,78^. Activated fibroblasts could thus promote recruitment of leucocytes and alter the behavior T-cells, via the upregulation of the SDF1 receptors CXCR4 and CXCR7 observed in AL, switching them from a migratory toward a stationary phenotype^80^. Such interactions between fibroblasts and infiltrated macrophages and CD4^+^ T-cells may thus maintain inflammation in AL.

This inflammatory positive feedback loop may also be sustained by crosstalk between macrophages and T-cells on the one hand, and T-cells and keratinocytes on the other hand. Indeed, M1 macrophages, coordinating a Th1 response, promote the infiltration and priming of T-lymphocytes towards proinflammatory Th1/Th17 CD4^+^ T-cells^29,76^. Consecutively as Th1 cells are known to secrete IFN-γ^81^, they may participate in the M1 macrophage polarization. Additionally, a co-stimulation between keratinocytes and T-cells could exist through the specific secretion of the T-cell-chemoattractant CCL27 by keratinocytes^47,48^, and the induction of the cytokine secretion and homeostasis disturbance of keratinocytes by CD4^+^ T-cells^82,83^.

**Figure 3:**
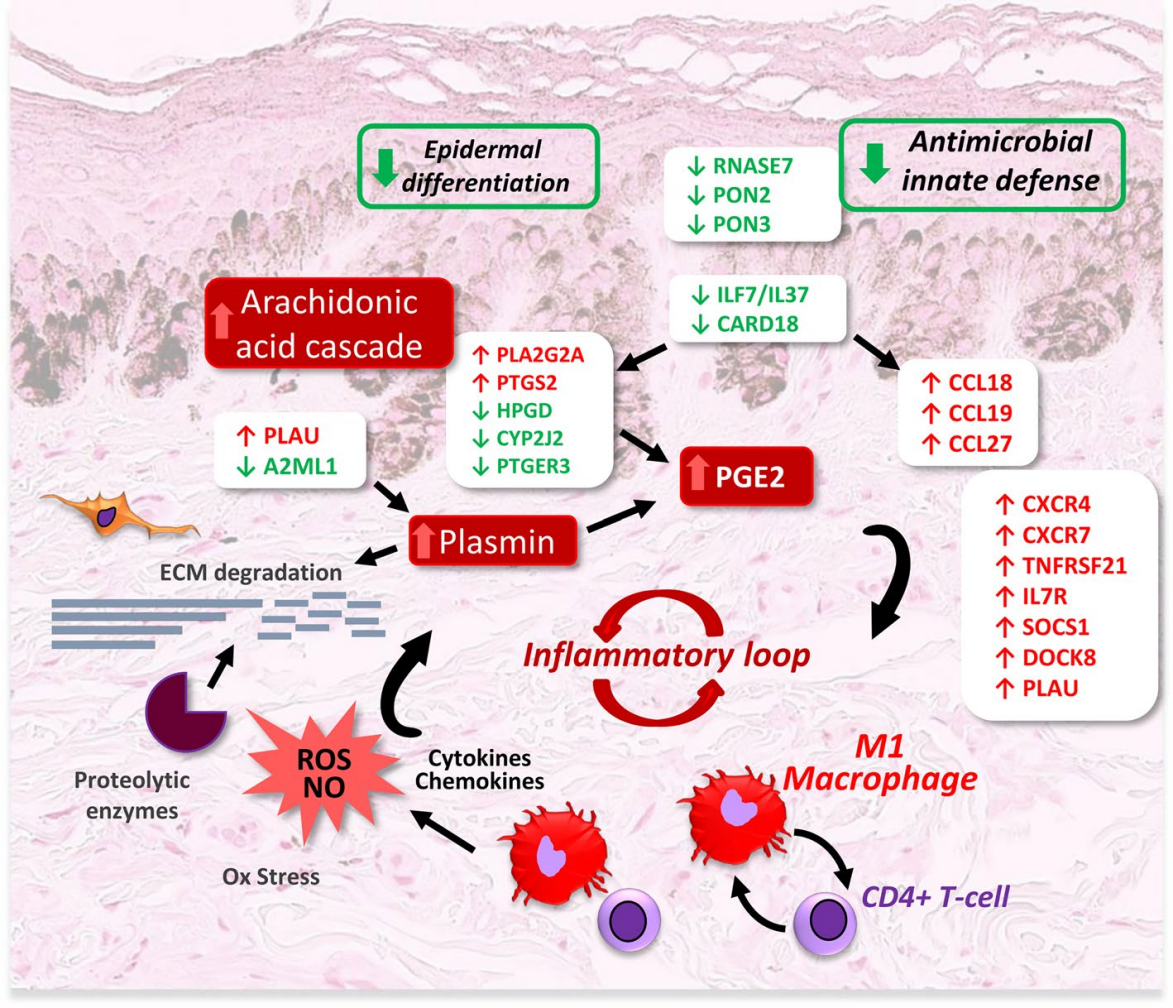
A pro-inflammatory loop sustains a chronic inflammation in actinic lentigines. The activation of pro-inflammatory pathways and the secretion of chemokines, prostaglandins and plasmin, as a consequence of the upregulation (red) or downregulation (green) of a large set of genes, may lead to the observed infiltration and activation of immune cells, CD4^+^ T-cells and pro-inflammatory M1 macrophages. In turn, these cells, notably M1 macrophages, secreting cytokines, reactive species and proteolytic enzymes, may have a deleterious impact on the skin cells and structure within the lesion. An inflammatory vicious circle thus takes place and leads to a chronic inflammation in actinic lentigines.

Altogether, the activation of key inflammatory signalings, the infiltration of pro-inflammatory macrophages and CD4^+^ T-cells, and the resulting production of cytokines, prostaglandins, chemokines, reactive species and proteolytic enzymes, may create an inflammatory self-perpetuating loop in AL, through cross-talks with skin cells (Fig. 3). As in the studied AL lesions, the melanogenesis pathway was not stimulated, this inflammatory vicious circle should not directly impact the activity of melanocytes but may lead to the establishment of a chronic low grade inflammation in the tissue. Long term consequences of this sustained sub-clinical inflammation may contribute to the alteration of the biological function and structure of the skin (epidermis and dermis), leading to abnormal epidermal invaginations and melanin accumulation.

The study highlights the infiltration of pro-inflammatory macrophages M1 as a hallmark of AL. To our knowledge, this is the first demonstration of M1/M2 imbalance in a skin hyperpigmented disorder. Initially recruited to the sites of injury, pro-inflammatory M1 macrophages can shift from M1 to M2 subtypes upon local signals, but a defective M1–M2 transition or dysfunctional macrophages can contribute to chronic inflammation.

Therefore, breaking this inflammatory vicious circle leading to chronic inflammation in AL, by using broad spectrum anti-inflammatory actives or promoting resolution of inflammation, could improve AL treatments.

## Methods

### Volunteers

Two open randomized prospective clinical studies have been performed, one included European women (France) (n = 15, aged 51–67 years; phototype II-III), and the other, Japanese women (n = 20, aged 54–71, phototype III-IV). The protocol complied with the Helsinki declaration and was approved by the local ethics committees (France: Comité de Protection des Personnes Sud Méditerranée V, ID 2007-A00175-48; Japan: Study N°503 approved by Nagoya City University of Medicine). Volunteers provided written informed consent.

### AL selection

AL from the dorsal side of the hands were selected through dermoscopic imaging (X70 magnification, Fotofinder dermoscope®, Teachscreen, Germany) and pigmented pattern scoring using the previously described methodology and criteria of selection^13,14^. Briefly, dermoscopic pictures of AL from European and Japanese volunteers, illustrated in Figure S3, show brown macules with irregular borders and demarcated pigmented patterns, from a regular, honeycomb-like structures (round pattern) to a network of loose and striated lines (elongated pattern). Only lesions containing at least 20% of elongated patterns determined using a dedicated software were selected for the European and Japanese studies.

### Processing of biopsies

Pairs of 3-mm biopsies (AL lesion and adjacent NL skin) were obtained from each volunteer. One set of biopsies (15 volunteers for the European study, 8 for the Japanese one) was embedded in paraffin for histology and immunostainings. A second set of skin biopsies (15 for the European study, 12 for the Japanese one) was dedicated to the transcriptomic study. The whole skin (epidermis and dermis) was processed for gene expression profiling using Affymetrix® U133A 2.0 chips (Affymetrix, USA) as described^13,14^.

### Microarrays data analysis*-*bioinformatics and statistical analysis

Raw data was normalized using the Robust Multichip Average method. Raw expression data have been deposited in NCBI’s Gene Expression Omnibus (GEO) database (accession number GSE192565). The generation of lists of differentially expressed genes between AL and NL in both studies has been reported^13,14^. Briefly, a mean fold change between AL and NL ≥ 1.5 for up-regulated genes or ≤ -1.5 for down-regulated genes and fdr adjusted *p* value at the 0.05 significance threshold. To generate the final list of common genes significantly modulated in both studies, the 178 probesets (136 genes) significantly modulated in the same way in AL in both studies with an absolute mean FC ≥ 1.5 were completed with probesets modulated in the same way in both populations, with an absolute mean FC ≥ 1.5 in one study and between 1.25 and 1.5 in the other (*p* value < 0.05) leading to a list of 245 common genes significantly modulated^14^.

To investigate upstream regulators and pathways, the list of common genes derived from statistical analyses, were exported into the Ingenuity Pathway Analysis Tool, IPA (Qiagen). Activated upstream transcription regulators were estimated by the z-score. The activation z‐score is used to infer likely activation states of upstream regulators based on comparison with a model that assigns random regulation directions. The *p* value of overlap indicates the statistical significance of genes in the dataset that are downstream of the upstream regulator. It measures whether there is a statistically significant overlap between the dataset genes and the genes that are regulated by a transcriptional regulator and is calculated using Fisher’s Exact Test. Upstream regulators were selected based on the two statistical measures: activation z core > 2 and an overlap *p* value < 0.01.

### Bibliographic study

To finely classify genes of interest into functional families, a bibliographic study was performed using Pubmed tools (http://www.ncbi.nlm.nih.gov/pubmed), with keywords related to skin, inflammation and/or immune cells. In case a gene or its corresponding protein had never been described in the context of skin biology, bibliographic study was enlarged without any key word and the most described function(s) of the gene or protein was retained.

### Validation of gene expression modulation assessed by quantitative PCR

Expression of genes found modulated in the microarray study was analyzed by quantitative PCR (qPCR). Complementary DNA (cDNA) was synthetized by reverse transcription of total RNA in presence of oligo(dT) and Transcriptor Reverse Transcriptase (Invitrogen, Thermo Fisher Scientific, USA). cDNA was then amplified using SsoAdvanced PreAmp Supermix (BIORAD). Q-PCR was performed using the LightCycler system (Roche Molecular System Inc., USA) and SYBR Green reagent mix (Ozyme, France) according to suppliers’ instructions. Primer sequences are detailed in Table S4. Glyceraldehyde-3-phosphate- dehydrogenase (GAPDH), beta-2- macroglobulin (B2M) and ribosomal protein S28 (RPS28) mRNA were quantified in each sample and used for normalization using Genorm application. For each volunteer, gene modulation was defined as the ratio between the level of mRNA expression in AL versus the level of mRNA expression in NL sample. For each gene, expression levels in AL samples versus expression levels in non-lesional samples were compared using Wilcoxon matched-pairs signed rank test (*p* < 0.05).

### Immunostaining of skin section

Immunolabelling was performed on formalin-fixed paraffin-embedded sections of 4µm thickness after heat-induced epitope retrieval with specific buffer (Target Retrieval Solution Tris/EDTA pH9 or Citrate pH6, Agilent Technologies, Santa Clara, USA). For enzymatic detection, endogenous peroxidase activity was blocked (Dual Endogenous Enzyme Block, Agilent Technologies, Santa Clara, USA) then incubation with primary antibodies was performed for 1h at room temperature. Staining was revealed by using horseradish peroxidase-conjugated secondary antibody polymers kit ImmPRESS (Vector Laboratories, Burlingame, USA) and 3-amino-9-ethylcarbazole was used as the chromogen (Agilent Technologies, Santa Clara, USA). The sections were briefly counterstained with haematoxylin before mounting. For immunofluorescence detection, primary antibodies were incubated for 1h at room temperature and revealed by a secondary antibody linked to a fluorescent chromophore. Cell nucleus was stained using DAPI (4’,6-diamidino-2-phenylindole).

Antibodies and supplemental technical treatment are given in the Table S5.

### Whole-slide scanning and image analysis

Brightfield images (enzymatic immunolabelled) and fluorescent images were acquired on NanoZoomer 2.0-HT (Hamamatsu Photonics, Hamamatsu, Japan) slide scanner for quantification. For illustration, fluorescent pictures were acquired on Slideview VS200 microscope (Olympus, Tokyo, Japan). Digital images were obtained from the entire 3 mm sample.

Quantification of the stained surface or cells was performed in the epidermis or the dermis. Measurement in the dermis was done to a depth of 500 µm from the dermal epidermal junction.

Bright field images were proceeded by Histolab 11.1 software (Microvision Instruments, Evry, France). A color image segmentation was applied to define positive pixels. For HLA-DR analysis, the surface of positive pixels was quantified to determine the surface of staining. The results were expressed by the surface of stained area per surface of epidermis or dermis. For all other markers, an automatic morphological operation (dilatation, erosion, size object) was added to gather pixels as entities (stained cells) and calculate the number of stained cells. Results are expressed as the number of stained cells per surface of the epidermis or the dermis.

Fluorescent images were proceeded using Image Fiji software. RGB Images were decomposed in three channels: blue for DAPI, green for CD68 staining, red for CD80, CD209 or CD163 staining. The cell nuclei were detected by automatic greythreshold intensity segmentation on the image obtained in the blue channel. Then, a binary image was created to determine a Region of Interest (ROI) around each nucleus expanding to 3µm. Each ROI was reported on green and red channels to make an analysis of particles and a selective threshold was applied to determine the positive pixels (grey level intensity above the threshold) in the green and red channels. ROIs with positive pixels in the green and red channels were considered as double positive cells. The number of positive cells CD68+ and double positive cells (CD80+/CD68+ or CD209+/CD68+ or CD163+/CD68+) per dermal surface were calculated for each AL and NL section for all the volunteers. Results are reported in a boxplot, the bottom and the top of the box represent the first and third quartiles, respectively and the horizontal line represents the median value.

Percentage of the number of double positive cells (CD80+/CD68+ or CD209+/CD68+ or CD163+ /CD68+) versus the total number of CD68+ cells for each section was also calculated. The means of the percentage (%) of AL and NL sections for all subjects (European and Japanese) are reported in pie charts.

### Statistics of immunostaining analysis

Statistic comparisons between NL and AL groups were carried out using paired Wilcoxon signed ranks test with a two-sided significance threshold set at 5%. All statistical analyses were performed using Prism Graph Pad software (Version 9; San Diego, California, USA). Analysis has been performed on 14 volunteers for the European study (systematical outlier results found for one volunteer have been subtracted) and on 8 volunteers for the Japanese study.

### Supplementary Information

Supplementary Information.

## Data Availability

Transcriptomic raw data have been deposited in NCBI’s Gene Expression Omnibus (GEO) database (series GSE192565). Other data are available from the corresponding author on reasonable request.
