# Supplementary material for: A chronic pro-inflammatory environment contributes to the physiopathology of actinic lentigines
Source: Sci Rep. 2024 Mar 4;14:5256. doi: 10.1038/s41598-024-53990-5 (PMC10912228; doi:10.1038/s41598-024-53990-5)
Supplement: Supplementary file 1 — Supplementary Information. [file 41598_2024_53990_MOESM1_ESM.pdf]

SUPPLEMENTARY INFORMATION

"A chronic pro-inflammatory environment contributes to the physiopathology of actinic lentigines"-
Christine Duval, Emilie Bourreau, Emilie Warrick, Philippe Bastien, Stéphanie Nouveau and Françoise Bernerd.

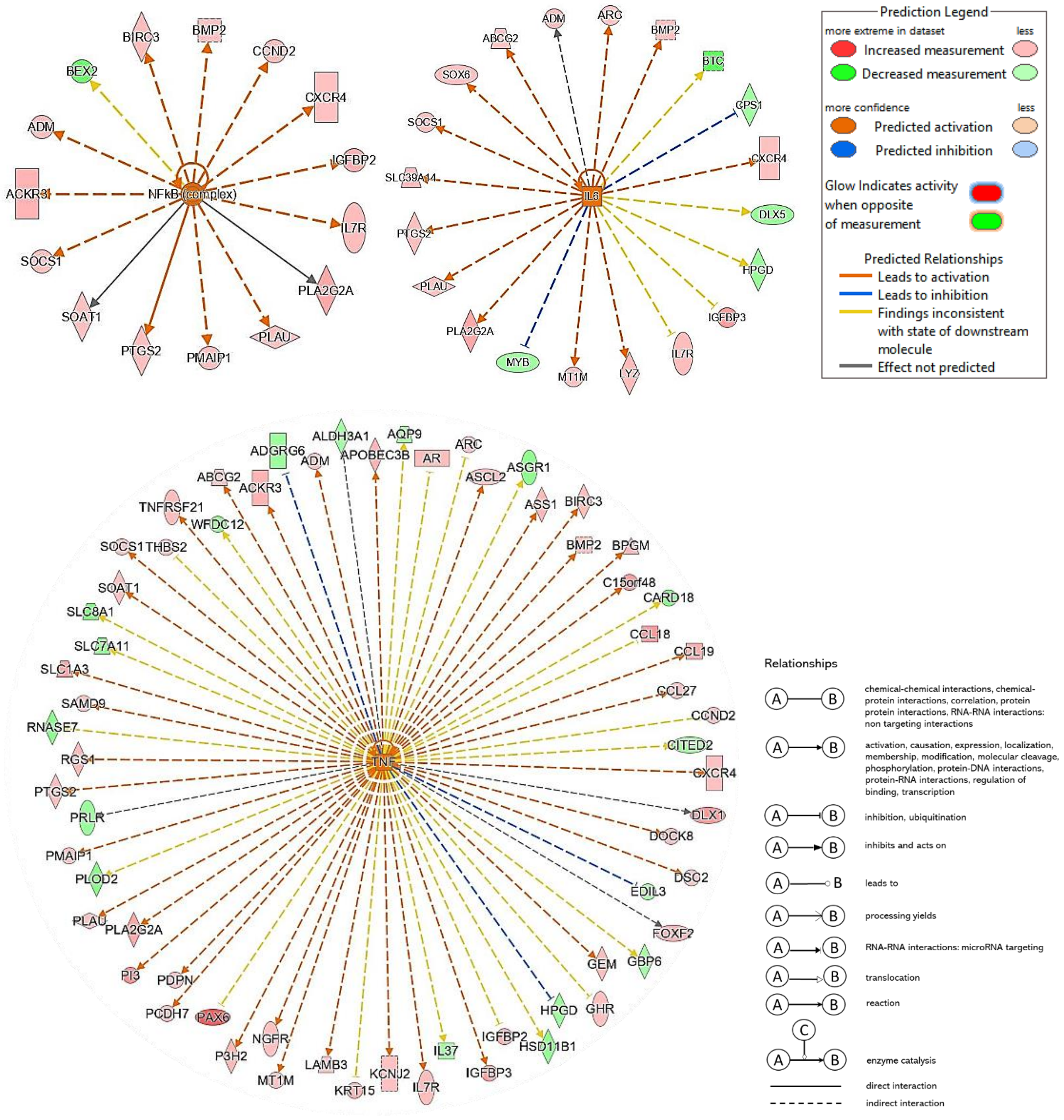

Suplementary Figure S1. Dataset targeted molecules of NFkB complex, TNF and Il-6 upstream regulators. My pathway (IPA) visualization of the interaction between 3 main upstream regulators NFkB (complex), IL6 and TNF, found from the list of genes modulated both in the European and Japanese AL, and their downstream targeted molecules in the dataset. NFkB complex interacts with 15 molecules in the dataset, Il-6 with 20, and TNF with 64.

Japan study

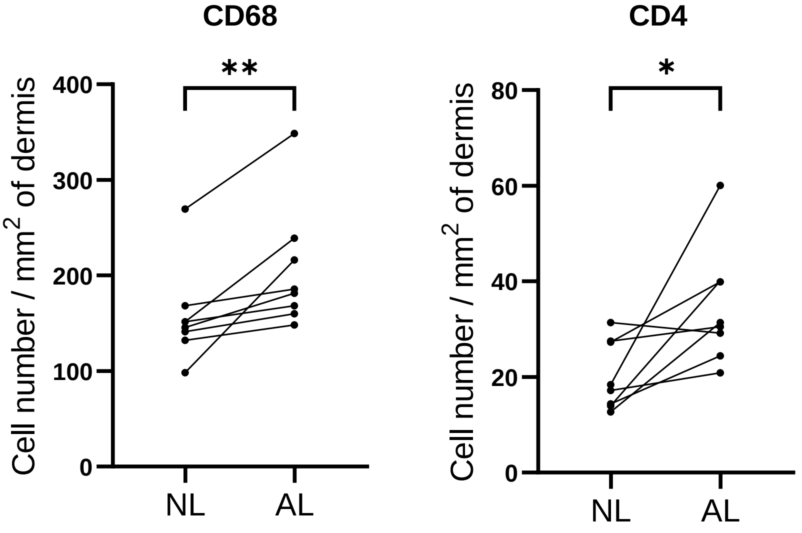

Supplementary Figure S2. Quantification of the CD68 and CD4 positive cells in AL and NL in the Japanese study.

Plots of CD68+ or CD4+ cells/mm^2 quantified by image analysis in the dermis of AL and NL for each volunteer from the Japanese study (n=8). Statistic comparisons between NL and AL groups were carried out using paired Wilcoxon signed ranks test: **P<0.01; *P<0.05

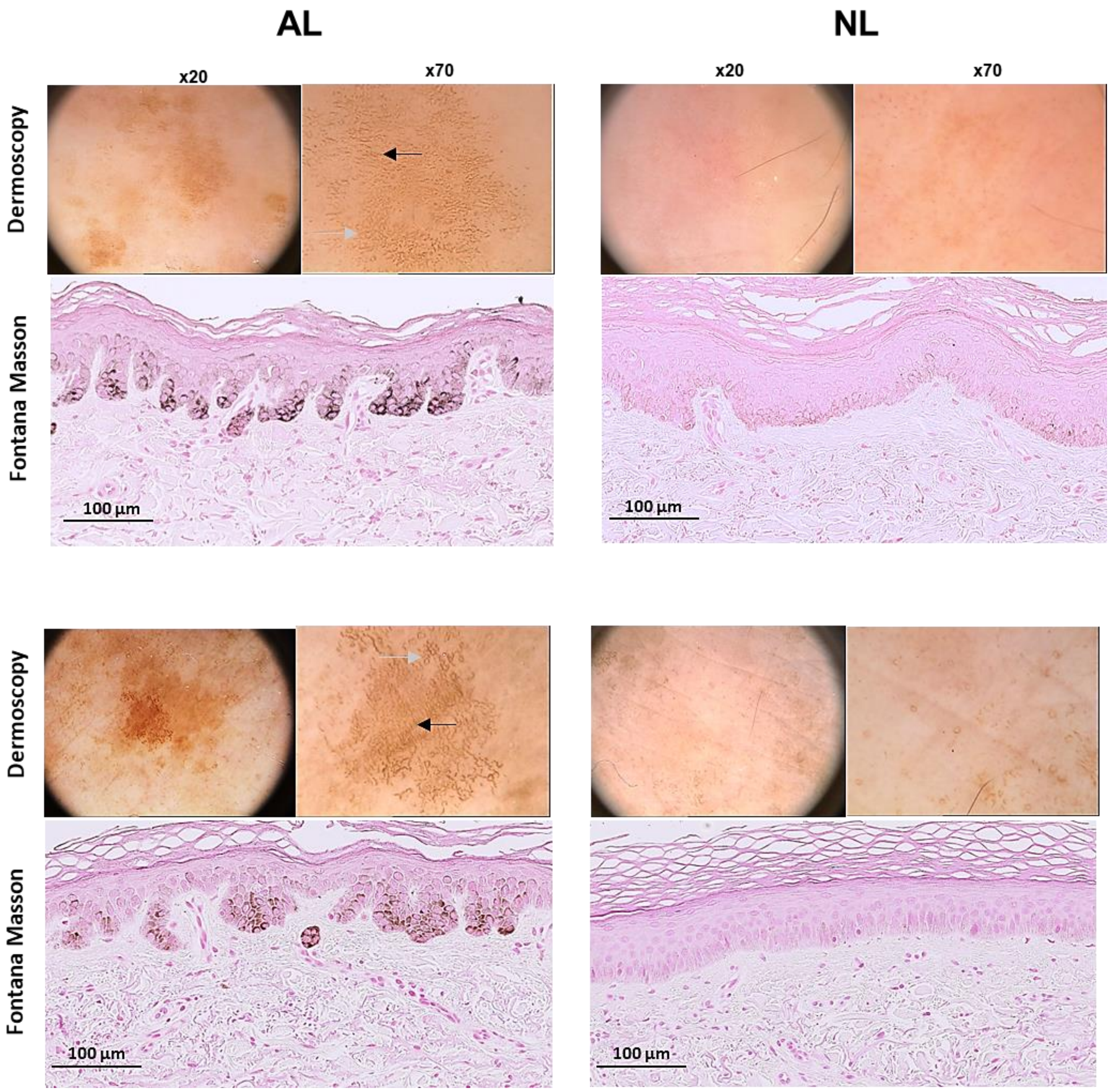

Supplementary Figure S3. Dermoscopic pictures and histological illustrations of actinic lentigines (AL) and non lesional skin (NL).

Dermoscopic images at x 20 and x70 and representative Fontana Masson-stained sections of AL and adjacent NL zones from a European volunteer (E48) and a Japanese one (J3). Dermoscopic pictures show brownish macules with irregular borders and sharply demarcated pigmented patterns, from a regular, honeycomb-like structures (round pattern, grey arrow) to a network of loose and striated lines giving a fingerprint appearance (elongated pattern, black arrow). Fontana Masson staining reveals the accumulation of melanin in the epidermis of AL, mainly in the basal layer and within the epidermal invaginations. No melanin accumulation was visible in the dermis.

Supplementary Table S1. Comparison of gene expression modulation assessed by quantitative PCR or microarray analysis in volunteers from Japan and Europe studies.

	PTGS2		CCL19		CCL27		PLAU		ADM		PON2		CARD18	
Ratio AL/NL	**RT-qPCR**	**microarrays**	**RT-qPCR**	**microarrays**	**RT-qPCR**	**microarrays**	**RT-qPCR**	**microarrays**	**RT-qPCR**	**microarrays**	**RT-qPCR**	**microarrays**	**RT-qPCR**	**microarrays**
**Japan study**														
J9	2.53	2.30	2.46	2.04	1.22	1.42	1.78	1.47	1.99	1.77	0.43	0.47	0.37	0.45
J10	3.55	1.36	0.73	2.27	0.73	1.64	16.93	2.53	3.43	1.09	5.60	0.51	0.27	0.56
J12	3.74	2.04	2.65	2.05	2.55	1.93	2.11	1.10	1.63	1.41	0.76	0.74	0.39	0.38
J13	0.49	0.46	2.80	2.33	1.60	1.58	1.97	1.63	2.43	1.90	0.52	0.54	0.40	0.45
J14	1.29	1.31	3.13	2.96	1.32	1.30	1.77	1.51	1.19	1.32	0.85	0.80	0.87	0.74
J15	1.93	2.94	2.42	2.66	1.78	1.71	1.09	1.18	1.28	1.36	0.57	0.67	0.42	0.42
J16	3.72	2.46	3.07	2.44	1.73	1.51	1.94	1.71	1.17	1.38	0.55	0.39	0.40	0.34
J18	4.93	2.21	1.67	1.43	1.56	1.43	1.62	1.24	4.03	2.81	0.66	0.48	0.34	0.36
J19	0.70	1.30	2.48	2.41	1.63	1.70	1.08	1.52	1.32	1.43	0.28	0.51	0.52	0.45
J20	2.50	2.37	1.89	1.31	2.92	2.69	2.06	1.84	2.99	1.59	0.53	0.50	0.51	0.44
**Geo. Mean**	**2.05**	**1.69**	**2.18**	**2.13**	**1.60**	**1.66**	**2.10**	**1.53**	**1.94**	**1.55**	**0.69**	**0.55**	**0.43**	**0.45**
GEO mean (all volunteers)		***1.57***		***2.06***		***1.51***		***1.47***		***1.57***		***0.58***		***0.48***
**Europe study**	0.74	1.04	3.52	2.87	1.13	1.10	1.69	2.14	2.95	2.36	0.59	0.62	0.50	0.43
E1	1.23	1.05	4.14	4.08	1.33	1.66	1.13	1.39	1.82	1.96	0.49	0.65	0.35	0.36
E2	1.70	2.16	1.68	1.46	0.74	0.75	1.44	1.75	1.37	1.69	0.54	0.62	0.94	0.95
E4	1.63	1.87	3.88	3.39	1.54	2.08	1.71	1.69	1.70	1.85	0.51	0.68	0.41	0.56
E5	4.90	2.38	1.51	1.16	2.14	1.77	2.53	2.13	2.24	1.66	0.60	0.58	0.69	0.60
E6	1.68	3.78	1.34	0.88	1.84	1.80	1.67	1.68	0.94	1.06	0.56	0.74	0.47	0.55
E7	1.81	1.75	7.78	6.73	0.97	1.20	1.84	1.91	3.18	2.07	0.61	0.57	0.49	0.58
E8	0.54	0.73	3.32	3.20	3.52	2.93	1.91	1.83	1.05	1.04	0.47	0.56	0.36	0.42
E9	2.41	1.56	5.96	3.16	1.67	1.63	2.85	2.01	7.63	4.06	0.64	0.54	1.25	1.05
E13	1.71	1.68	2.20	2.38	1.81	2.38	0.72	0.84	1.71	1.47	0.24	0.32	0.22	0.41
**Geo. Mean**	**1.56**	**1.63**	**3.03**	**2.50**	**1.53**	**1.62**	**1.64**	**1.69**	**2.02**	**1.79**	**0.51**	**0.58**	**0.51**	**0.56**
GEO mean (all volunteers)		***1.55***		***1.79***		***1.49***		***1.56***		***1.65***		***0.61***		***0.58***

Supplementary Table S1. Comparison of gene expression modulation assessed by quantitative PCR or microarray analysis in volunteers from Japan and Europe studies. The expression of genes that were found significantly modulated in both microarray studies and representative of the inflammation/ immune response subfamilies, was analysed by quantitative PCR in 10 volunteers from each study, Japanese (Jnb) and European (Enb). Ratios of modulation in AL versus NL skin were calculated for each volunteer and compared to those obtained through microarray analysis. Cells in red indicate ratio values ≥ 1,5 and cells in green indicate ratio values ≤ 0,67. With few exceptions, a strong correlation was found between qPCR and microarray results for all volunteers. Geo. mean: geometric mean of ratios from 10 volunteers. Bold mean indicates a significant difference between AL and NL samples ($p < 0.05$, paired Wilcoxon signed ranks test). Geo. Mean (all volunteers): the geometric mean of ratios from all volunteers (n=12 for Japan study, n=13 for Europe study) from microarray studies is indicated in italic.

Supplementary Table S2. Comparison of CD68 and CD4 positive cells in AL and NL in the European and Japanese studies.

	Europe		Japan	
**Cell nb /mm2**	**FC AL/NL Mean**	***pvalue***	**FC AL/NL Mean**	***pvalue***
**CD68+**	1.96	*0.013*	1.35	*0.008*
**CD4+**	2.66	*0.009*	1.87	*0.016*

Supplementary Table S2. Comparison of CD68 and CD4 positive cells in AL and NL in the European and Japanese studies.

The fold change (FC) of the number of CD68+ or CD4+ cells per mm2 of dermis between AL and NL has been calculated for each volunteer from the European (n= 14) and Japanese (n=8) studies. The means of the fold change (FC) are indicated. The comparison of the number of CD68+ or CD4+cells/mm2 between AL and NL has been assessed using paired Wilcoxon signed ranks test: p-value < 0.05 indicates significant difference.

Supplementary Table S3. Quantification of macrophages subsets in AL and NL in the European and Japanese studies.

	Europe			Japan		
	Mean of cells number/mm^2		*pvalue*	Mean of cells number/mm^2		*pvalue*
	NL	AL		NL	AL	
CD68+CD80+	3.2+/- 5.6	34+/- 46	*0.0007*	16+/-14	48+/-31	*0.032*
CD68+CD209+	60+/- 40	81 +/-57	*0.110*	52+/-35	91+/-75	*>0.999*
CD68+CD163+	113+/- 84	179+/- 139	*0.123*	102+/-48	103+/-59	*0.1484*

Supplementary Table S3. Quantification of macrophages subsets in AL and NL in the European and Japanese studies. The number of CD68+/CD80+cells, CD68+/CD209+cells and CD68+/CD163+cells per mm2 (mean +/-SD) in NL and AL form the European and Japanese studies, are reported. The comparison has been assessed using paired Wilcoxon signed ranks test: p-value < 0.05 indicates significant difference. A significant increase of the number of CD68+/CD80+cells in AL versus NL for both studies is revealed.

Supplementary Table S4. PCR primer sequences

Gene	Symbol	Gene Bank	sense primer	antisense primer
Glyceraldehyde-3-phosphate dehydrogenase	GAPDH	NM_002046	GGCTCTCCAGAACATCATCCCTGC	GGGTGTCGCTGTTGAAGTCAGAGG
Ribosomal protein S28	RPS28	NM_001031	CCGTGTGCAGCCTATCAAG	CAAGCTCAGCGCAACCTC
Beta-2-microglobulin	B2M	NM_004048	TTTCATCCATCCGACATTGA	CCTCCATGATGCTGCTTACA
Chemokine (C-C motif) ligand 19	CCL19	NM_006274	GGAGTCCGAGTCAAGCATTG	GGTAGCATTGCAATCTGGGG
Prostaglandin-endoperoxide synthase 2	PTGS2	NM_000963	TGAGCATCTACGGTTTGCTG	TGCTTGTCTGGAACAACTGC
Plasminogen activator, urokinase	PLAU	NM_002658	GGGTGGTCCTGACTCAACAT	TTGGCTAAGCTCCCTCAAGA
Paraoxonase 2	PON2	NM_001018161, NM_000305	CATGAGCCAATATGTCAGCAACA	CTGTTGGACCGGCACATTTC
Caspase recruitment domain family, member 18	CARD18	NM_021571	CTTTGAGGCAAGTTGAGGGTC	GCTCGAGTCTTGATTGACCTTG
Chemokine (C-C motif) ligand 27	CCL27	NM_006664	CAGCTCTACCGAAAGCCACT	GCATCCCAAAATTCAGCTTG
Adrenomedullin	ADM	NM_001124	CTTATTCGGCCCCAGGACAT	ACTGGTAGATCTGGTGTGCC

Supplementary Table S5. Antibodies and technical treatment for immunochemistry

Primary Antibody	Supplier	Reference	Clone	Host/ Isotype	Dilution	Antigen retrieval method
Anti-HLA-DR	DAKO (Glostrup. Denmark)	M0746	TAL.1B5	Mouse IgG1	1/40	Citrate Buffer PH6 - 20min 95°C
Anti-CD68	DAKO (Glostrup. Denmark)	M0876	PG-M1	Mouse IgG3	1/50	Citrate Buffer PH6 preboiled by microwave 40 min in cooling buffer
Anti-CD4	DAKO (Glostrup. Denmark)	M7310	4B12	Mouse IgG1	1/50	20min 95°C-Citrate Buffer PH6
Anti-CD8	DAKO (Glostrup. Denmark)	M7103	C8/144B	Mouse IgG1	1/70	20min 95°C-Tris Buffer PH9
Anti-CD117	Leica Biosystems (Newcastle. UK)	NCL-L-CD117-032	T595	Rabbit IgG	1/5	40min 95°C-Tris Buffer PH9
Anti-CD11c	AbCam (Cambridge. UK)	Ab52632	EP1347Y	Rabbit	1/50	20min 95°C-Citrate Buffer PH6
Anti-Elastase	DAKO (Glostrup. Denmark)	M0752	NP57	Mouse IgG1	1/100	No retrieval
Anti-CD80	Origene (Rockville. USA)	TA501576S	OTI2E5	Mouse IgG2a	1/100	Citrate Buffer PH6 preboiled by microwave 40 min in cooling buffer
Anti-CD209	BD Technologies (NC. USA)	551249	DCN46	Mouse IgG2b	1/50	Citrate Buffer PH6 preboiled by microwave 40 min in cooling buffer
Anti-CD163	Bio-Rad AbD Serotec (Oxford. UK)	MCA1853	EDHu-1	Mouse IgG1	1/600	Citrate Buffer PH6 preboiled by microwave 40 min in cooling buffer

Secondary Antibody	Supplier	Reference	Dilution
Goat anti-Mouse IgG3 Cross-Adsorbed Secondary Antibody, Alexa Fluor™ 488	Invitrogen (Carlsbad, USA)	A-21151	1/200
Goat anti-Mouse IgG1 Cross-Adsorbed Secondary Antibody, Alexa Fluor™ 594	Invitrogen (Carlsbad, USA)	A-21125	1/200
Goat anti-Mouse IgG2a Cross-Adsorbed Secondary Antibody, Alexa Fluor™ 594	Invitrogen (Carlsbad, USA)	A-21135	1/200
Goat anti-Mouse IgG2b Cross-Adsorbed Secondary Antibody, Alexa Fluor™ 594	Invitrogen (Carlsbad, USA)	A-21145	1/200
